# The Utility of Baseline Characteristics and [^123^I]MIBG Cardiac Adrenergic System Scintigraphy in Qualifying Patients with Post-Infarction Heart Failure for Implantable Cardioverter-Defibrillator (ICD) Placement

**DOI:** 10.3390/jcm13216378

**Published:** 2024-10-24

**Authors:** Anna Teresińska, Aneta Fronczak-Jakubczyk, Olgierd Woźniak, Aleksander Maciąg, Jarosław Jezierski, Alicja Cicha-Mikołajczyk, Piotr Hoffman, Elżbieta Katarzyna Biernacka

**Affiliations:** 1 Department of Nuclear Medicine, Institute of Cardiology, 04-628 Warsaw, Poland; ateresinska@ikard.pl; 2 Department of Arrhythmia, National Institute of Cardiology, 04-628 Warsaw, Poland; aneta.fronczak@gmail.com (A.F.-J.); jsjezierski65@tlen.pl (J.J.); 3 Department of Congenital Heart Diseases, National Institute of Cardiology, 04-628 Warsaw, Poland; 4 Department of Epidemiology, Cardiovascular Diseases Prevention and Health Promotion, National Institute of Cardiology, 04-628 Warsaw, Poland

**Keywords:** scintigraphy, heart failure, ICD

## Abstract

**Background:** Post-infarction heart failure with reduced ejection fraction (HFrEF) patients often face persistent risks of sudden cardiac arrest leading to sudden cardiac death. While implanting a cardioverter-defibrillator (ICD) can enhance prognosis, complications and costs limit its widespread use. Current patient qualification criteria, relying on imperfect parameters, require refinement. The impairment of the cardiac adrenergic system in heart failure is associated with ventricular arrhythmias. The goal of the study was to assess the utility of cardiac adrenergic system scintigraphy in qualifying patients for ICD placement. **Methods:** In this prospective study of 85 post-infarction HFrEF patients at a single center, clinical assessments, laboratory tests, echocardiography, [^123^I]MIBG scintigraphy, and ICD implantation were performed. Scintigraphy involved planar chest images and evaluating the heart-to-mediastinum ratio (H/M) and washout rate (WO). SPECT imaging assessed [^123^I]MIBG uptake in 17 left ventricular segments to calculate the summed difference score (SDS). **Results:** During a median of 4-year follow-up, 22% of patients experienced appropriate ICD interventions, and 25% of patients died or underwent heart transplantation. The mean values of analyzed parameters did not significantly differ between groups. In the univariate analysis, younger age and moderately impaired left ventricular ejection fraction (LVEF) were correlated with more frequent ICD interventions. In comparison, older age and elevated NT-proBNP levels were associated with death or heart transplantation. Additionally, the univariate analysis identified SDS-15′ as a prognostic factor for death/heart transplant. The multivariate analysis identified predictors for ICD interventions, including younger age, an EF of 30% or greater, and a larger left ventricular end-diastolic diameter. In contrast, older age and an LVEF of less than 25% were significant predictors of death or heart transplantation. **Conclusions:** Scintigraphic parameters did not effectively predict ICD interventions or death/heart transplantation, though the summed difference score demonstrated potential as a prognostic factor. Younger age with moderately impaired EF correlated with frequent ICD interventions, while in older age, EF < 25% predicted death or transplantation. Further investigation is needed for patients with borderline EF values.

## 1. Introduction

Heart failure (HF) has been recognized as an emerging pandemic. It affects 64 million individuals globally [1], and its prevalence rises with advancing age, accounting for a significant portion of hospitalizations in the group of patients over 75 years of age. In recent decades, we have observed a reduction in the age-standardized morbidity rate, which reflects progress in the diagnosis and treatment of HF. Nevertheless, due to society’s aging and the increase in the number of older individuals, the absolute number of HF patients is rising.

Medical treatment for HF patients is based on several disease-modifying drugs that can reduce mortality and improve quality of life. In some patients, despite appropriate treatment, sudden cardiac arrest (SCA) occurs, which, without immediate intervention and implementation of a cardiopulmonary resuscitation (CPR) protocol, leads to sudden cardiac death (SCD) [2]. In SCA resulting from ventricular tachycardia (VT) or ventricular fibrillation (VF), the time from the onset of arrhythmia to defibrillation is crucial [3]. Implantation of a cardioverter-defibrillator system (ICD) significantly shortens this time, thus improving the prognosis for patients.

The use of ICDs is limited to high-risk SCD patients due to complications and costs. [4]. This group includes SCA survivors (secondary prevention) and patients with post-myocardial infarction HF, structural heart diseases, or inherited arrhythmia syndromes (primary prevention). While ICDs significantly reduce mortality in secondary prevention [5,6,7,8], this group is a minority. While ICDs significantly reduce mortality in secondary prevention, this group is a minority. Despite advancements, identifying other at-risk individuals remains challenging, with many analyses focusing on post-myocardial infarction HF patients [2].

Current criteria for ICD qualification are based on heart failure symptoms and left ventricular ejection fraction (EF), but they have limitations. Only about 35% of ICD patients experience interventions, while some individuals with higher EF remain at risk of sudden cardiac death (SCD). Additionally, roughly two-thirds of SCD cases associated with coronary heart disease occur either as the first clinical manifestation of coronary heart disease or in individuals categorized as low-risk by existing risk prediction methods [2]. This demonstrates the need to optimize the adopted criteria.

The impairment of cardiac muscle function observed in heart failure (HF) activates both local and systemic compensatory mechanisms. In the early stages of HF, these mechanisms help maintain cardiac output adequate for demand, thereby preventing the clinical manifestation of HF. The compensatory response is primarily achieved through increased activation of the adrenergic system. Sympathetic fibers innervate the conduction system and myocardium, modulating heart rate, and contractility to adjust them to the current demand. During short-term cardiovascular system overload, these mechanisms enable a physiological response in the heart. However, over time, they become an additional burden on the already strained cardiac muscle, leading to the development of a ‘vicious cycle’ and further progression of HF. As the disease advances, permanent remodeling of the cardiac muscle occurs. Cardiac denervation is an adaptive mechanism to this state. As a result, the muscle becomes less responsive to increased sympathetic activation, which slows down the pathophysiological cascade driving disease progression.

Sympathetic stimulation depends on norepinephrine (NE), which is stored in sympathetic nervous system axons within the heart. NE is released during nerve activation, stimulating β-adrenergic receptors on cardiomyocytes and triggering contractions. This process is essential for the heart’s ability to adapt to various physiological demands, particularly during periods of increased stress or physical exertion. However, the prolonged release of NE can lead to a range of complications, including increased heart rate and contractility, which may ultimately contribute to cardiac dysfunction. Metaiodobenzylguanidine (MIBG) is a chemical NE analog that enables the assessment of presynaptic sympathetic innervation integrity. After labeling MIBG with the iodine-123 radioisotope, scintigraphy allows for the visualization of abnormalities in both global and regional adrenergic innervation of the heart. This imaging technique is crucial for understanding the state of cardiac sympathetic innervation and its correlation with heart failure progression.

By acquiring planar images, it is possible to assess global cardiac adrenergic innervation. This can provide essential insights into the overall sympathetic function of the heart. Beyond the global impairment of adrenergic innervation in the cardiac muscle, the prognosis may also depend on the location of innervated areas and their regional intensity, which can be assessed using three-dimensional SPECT (Single Photon Emission Computed Tomography) imaging. Regional assessments are particularly valuable in identifying specific areas of the heart that may be at risk for arrhythmias due to impaired innervation.

NT-proBNP is a biomarker that reflects cardiac stress and dysfunction, and its measurement provides valuable information on patient prognosis, risk stratification, and the degree of compensation in patients with heart failure. Monitoring NT-proBNP can ease and guide treatment decisions and evaluate the effectiveness of therapeutic interventions.

The goal of the study was to assess the utility of cardiac adrenergic system scintigraphy in qualifying patients with post-infarction heart failure for implantable cardioverter-defibrillator (ICD) placement.

## 2. Methods

This is a prospective single-center study with patient recruitment spanning from 2013 to 2016. The study population consisted of consecutive patients with post-infarction heart failure (HF) who were qualified for implantable cardioverter-defibrillator (ICD) or cardiac resynchronization therapy with defibrillator (CRT-D) implantation based on the currently applicable guidelines from the European Society of Cardiology (ESC) for the diagnosis and treatment of acute and chronic heart failure, published in 2008 [9] and 2016 [10], depending on the patient’s hospitalization date).

The criteria for primary prevention of sudden cardiac death (SCD) are summarized in Table 1.

The study was approved by the Local Bioethics Committee of the Institute of Cardiology. All participants provided informed and signed consent prior to their recruitment in the study.

### 2.1. Scintigraphy

A non-invasive radioisotope examination using metaiodobenzylguanidine labeled with radioactive iodine ([^123^I]MIBG) was conducted within a maximum of 3 months relative to ICD or CRT-D implantation. The examination was performed according to a standardized protocol using a dual-head gamma camera (AXIS, Picker/Philips) equipped with low-energy high-resolution (LEHR) collimators, employing both planar and single-photon emission computed tomography (SPECT) techniques.

None of the patients recruited for the study were taking medications that could affect MIBG uptake. Each participant was instructed to refrain from eating food or taking compounds that could influence MIBG distribution for at least 24 h before the MIBG injection. Prior to the intravenous administration of the radioisotope, each patient received 400 mg of sodium perchlorate to block thyroid uptake.

Global MIBG cardiac uptake was evaluated using planar images of the heart acquired from an anterior thoracic view, 15 min (early studies) and 3.5 h (late studies) after MIBG injection. The analyzed parameters were: (1) the heart-to-mediastinum ratio (H/M ratio) (cardiac MIBG uptake relative to a mediastinal reference uptake, where ‘H’ represents the mean count per pixel in the heart and ‘M’ represents the mean count per pixel in the upper mediastinum) for both early and late studies; and (2) the washout rate (WR), calculated using the formula: (early H/M ratio − late H/M ratio) ÷ early H/M ratio × 100 (%).

SPECT image acquisitions began immediately after the completion of planar studies. Projections were acquired from the right anterior oblique to the left posterior oblique view. The data were then reconstructed using filtered back projection (FBP). Following this, the quality of the obtained images was assessed. Studies with excessively high extracardiac uptake, preventing an accurate assessment of tracer uptake by the heart, were deemed non-diagnostic and were not included in further analysis. In the remaining studies, a semi-quantitative assessment of [^123^I]MIBG uptake was conducted. [^123^I]MIBG uptake was evaluated in each of the 17 left ventricular segments using the following scale: 0—normal uptake, 1—minimal uptake impairment, 2—slight uptake impairment, 3—significant uptake impairment, and 4—no uptake. The total summed defect score (SDS) was obtained by summing the impairment scores across individual segments in early (SDS-15′) and late (SDS-4h) SPECT imaging.

### 2.2. Echocardiographic Examination

Echocardiographic examinations (ECHO) were conducted using Vivid S70 and Vivid S95 machines (General Electric) according to the European Association of Cardiovascular Imaging (EACVI) standards, either before ICD implantation or within a week post-implantation. The following parameters were assessed: global left ventricular ejection fraction (LVEF) using the biplane Simpson’s method, left ventricular end-diastolic diameter in the parasternal long-axis view, and regional wall motion abnormalities.

### 2.3. NT-proBNP

N-terminal pro-b-type natriuretic peptide (NT-proBNP) [pg/mL] levels were measured once in each patient admitted to the hospital during the hospitalization in which they were evaluated for ICD implantation. This single measurement was taken as part of the standard diagnostic protocol to assess heart failure status.

### 2.4. Follow-Up

Patients were followed up for a minimum of two years. Regular visits were scheduled every 6 months, during which patients underwent monitoring for ICD therapies, including anti-tachycardia pacing or high-energy therapy for sustained ventricular tachycardia (VT) or ventricular fibrillation (VF), and were screened for clinical endpoints (death due to heart failure or heart transplant). The confirmation of appropriate ICD therapy was based on the analysis of stored electrograms.

### 2.5. Statistical Analysis

Statistical analysis was carried out using SAS version 9.4 software (SAS, Cary, NC, USA). Data were presented as mean (SD) or median (IQR) for quantitative variables and as number (percentage) for qualitative variables. The normality of the distribution of analyzed variables was tested using the Shapiro-Wilk test. The comparison of the distribution of values for quantitative variables between subgroups with and without the event was performed using the Student’s *t*-test (for normally distributed variables) and the non-parametric Mann-Whitney test (for non-normally distributed variables). Qualitative variables were compared using the χ^2^ test and Fisher’s exact test.

To determine independent predictors of the endpoint (appropriate ICD therapy or death due to heart failure/heart transplant), both univariate and multivariate Cox regression analyses were used. The multivariate analysis included parameters whose association with the endpoint was clinically relevant or achieved statistical significance in univariate analysis with a *p*-value < 0.1. Diagnostic accuracy for predicting the endpoint was presented as sensitivity and specificity. The level of significance was set at *p* < 0.05, while *p*-values between 0.05 and 0.10 were considered to indicate a statistical trend.

## 3. Results

### 3.1. Characteristics of the Study Group

The clinical characteristics of the studied group are presented in Table 2. The study included 85 patients (87% male, age 66.4 ± 8.4 years, EF 27.2% ± 5.3) with post-infarction heart failure who were qualified for ICD system implantation at the Institute of Cardiology in Warsaw between 2013 and 2016 and who were followed for a median of 4 years.

The studied population exhibited a high prevalence of typical coronary artery disease risk factors: arterial hypertension (61% of patients), smoking (34% of patients), and diabetes (26% of patients). The majority of patients had experienced one myocardial infarction (70% of patients). A history of two or three myocardial infarctions was documented in 30% of patients. Percutaneous coronary intervention (PCI) was performed in 74% of patients (20% underwent the procedure twice, 8% three times, and one patient underwent the procedure six times). In comparison, 21% of patients underwent coronary artery bypass grafting.

All patients included in the study had symptoms of heart failure (NYHA II-IV), with the majority (74%) classified as NYHA functional class II.

The indications for ICD system implantation in 89% of patients were for the primary prevention of sudden cardiac death (SCD). In the remaining 11% of patients, ventricular fibrillation (VF) or hemodynamically intolerant ventricular tachycardia (VT) without a reversible cause (excluding the first 48 h post-myocardial infarction) were documented. All patients included in the study had indications for ICD implantation for primary prevention of SCD, regardless of the occurrence of VF or intolerant VT.

### 3.2. Comparison of Patients with ICD Interventions with Patients without ICD Interventions

The study included 85 patients (87% male, mean age 66.4 ± 8.4 years, left ventricular ejection fraction 27.2% ± 5.3). In the studied population, antitachyarrhythmic pacing (ATP) and/or high-energy therapy occurred in 22% of patients (Table 2), with a median time from device implantation to the first adequate intervention of 2.3 years (range 1.2–5.2). Adequate ICD intervention occurred more frequently in younger patients (age 61.3 ± 8.5 years vs. 67.8 ± 7.8 years; *p* < 0.002) and in patients with moderately impaired left ventricular systolic function compared to those with severe left ventricular systolic dysfunction (i.e., patients with EF 30–35% compared to those with EF < 30%; *p* < 0.004). Trends toward statistical significance were observed when comparing patients without PCI, those with one intervention, and those with two or more interventions, with a more frequent occurrence of ATP/high-energy therapy in patients with multiple interventions (*p* < 0.09).

### 3.3. Comparison of Patients Who Survived the Observation Period with Those Who Died or Underwent Heart Transplantation

Patients who experienced death or heart transplantation were older (trend level significance: 65.4 ± 7.8 years vs. 69.3 ± 9.5 years; *p* < 0.06), had a statistically significantly lower resting heart rate (70.4 ± 11.1 bpm vs. 63.6 ± 8.2 bpm; *p* < 0.01), and higher levels of N-terminal pro-B-type natriuretic peptide (NT-proBNP) (6.9 [6.4–7.5] vs. 7.4 [6.9–8.2]; *p* < 0.02) (Table 3). The ejection fraction (EF) value was not lower in patients who experienced death or heart transplantation.

The group of patients in which death or heart transplantation occurred did not significantly differ from the other patients in terms of the scintigraphy values obtained from the cardiac adrenergic system using [^123^I]MIBG.

Patients were observed for an average of 57 months (range 3–93 months). During this period, 9% of patients experienced adequate ATP therapy, 13% experienced adequate defibrillation, heart transplantation was performed in one patient (1%), and 24% of patients died due to worsening heart failure (Table 4).

### 3.4. Univariate Analysis

**Figure 1:**
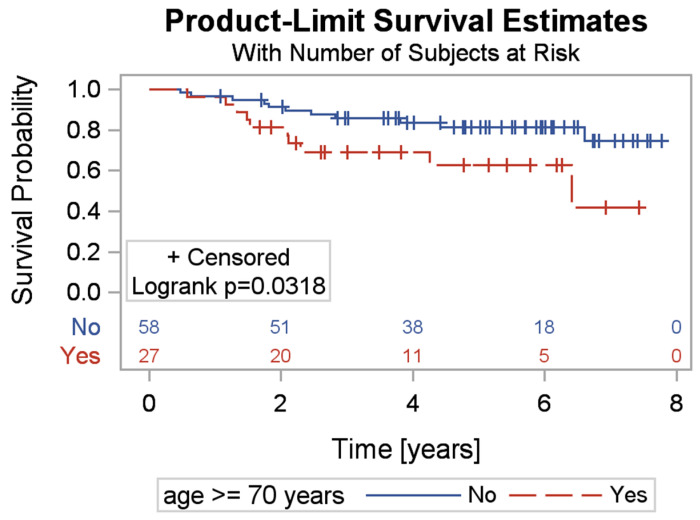
Comparison of survival in patients aged ≥70 years with patients <70 years.

A univariate analysis was conducted (Table 5). Age proved to be a predictor of both adequate ICD intervention and death or heart transplantation, with younger patients being at greater risk of ICD intervention and older patients at greater risk of death or transplantation (Figure 1).

**Figure 2:**
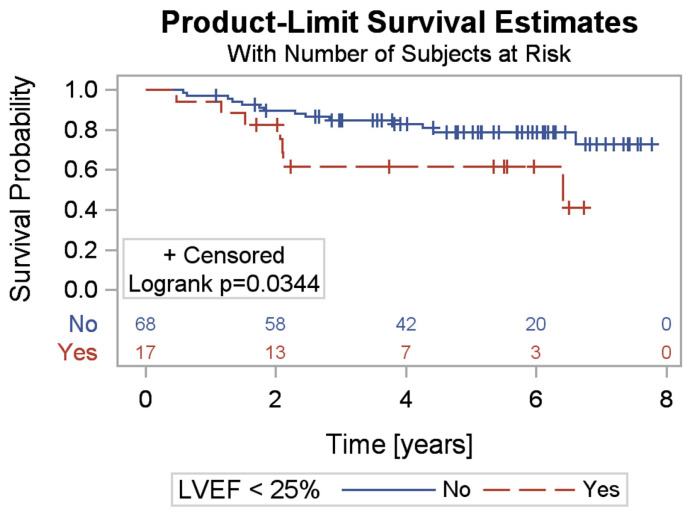
Comparison of survival in patients with LVEF < 25% and LVEF ≥ 25%.

An inverse relationship was also observed regarding left ventricular systolic function. Patients with an ejection fraction (EF) of 30–35% had a higher risk of ICD intervention, while patients with an EF < 25% had a higher risk of death or heart transplantation (Figure 2). Additionally, factors that worsened the prognosis included higher NT-proBNP levels and a lower resting heart rate (HR).

The risk of ICD intervention was higher in patients with a significantly enlarged left ventricular dimension (LVEDd ≥ 65 mm).

The value of the SDS parameter obtained in the early phase of [^123^I]MIBG scintigraphy was a predictor of death or heart transplantation in univariate analysis (HR 1.079; 95% confidence interval 1.007–1.157). It should be emphasized that this result was based on an analysis of only 62% of the cohort included in the study (with the quality of studies for the remaining patients assessed as “not suitable for analysis”). No predictive value was demonstrated for other parameters obtained during [^123^I]MIBG scintigraphy.

### 3.5. Multivariate Analysis

Multivariate analysis identified independent factors influencing the occurrence of death or heart transplantation: more advanced patient age (HR 1.062; 95% confidence interval 1.006–1.122; *p* = 0.031) and EF < 25% (HR 2.512; 95% confidence interval 1.004–6.286; *p* = 0.049) (Table 6).

An analysis was also conducted on factors influencing the occurrence of ATP or adequate ICD intervention. Independent factors included: younger age (HR 0.912; 95% confidence interval 0.846–0.983; *p* = 0.016); EF ≥ 30% (HR 4.632; 95% confidence interval 1.538–13.954; *p* = 0.006), and higher LVEDd value (HR 1.096; 95% confidence interval 1.017–1.181; *p* = 0.016) (Table 7).

Figure 1 compares the survival curve of patients under 70 years old with those aged 70 and older. The highest risk of death occurs in patients aged 70 and older (*p* = 0.03). A survival curve was also plotted for patients with EF < 25% and EF ≥ 25%. A significantly worse prognosis was observed in patients with EF < 25% (*p* = 0.03) (Figure 2).

## 4. Discussion

The study aimed to analyze the utility of [^123^I]MIBG cardiac adrenergic system scintigraphy in predicting the occurrence of appropriate ICD therapy and identifying patients at high risk of death or heart transplantation among those with post-infarction heart failure.

The results did not reveal a scintigraphic parameter that was significantly more frequent in patients who underwent ICD intervention. None of the analyzed parameters were effective in identifying patients at high risk of appropriate ICD intervention. The significance of predictors suggested by some researchers for appropriate ICD intervention was not confirmed (Table 8). It is worth noting that some authors used different endpoints than those in this study, such as a composite endpoint of NYHA functional class progression, potentially life-threatening arrhythmic events, or cardiac death (e.g., Jacobson). Others, such as Arimoto et al., examined a different cohort of patients with heart failure regardless of EF value.

A factor associated with an increased risk of ICD therapy was younger age. This finding aligns with the literature, which reports that the benefit of ICD decreases with age [16,17]. It is essential to emphasize that both younger and older patients benefit from ICD implantation; therefore, age alone should not determine ICD qualification.

Additionally, patients with a larger left ventricular end-diastolic dimension (LVEDd), assessed by echocardiography, had a higher risk of adequate ICD intervention. An enlarged left ventricular cavity may indirectly indicate deformation resulting from myocardial infarction. Within the scar and surrounding tissues, arrhythmias requiring ICD intervention may develop, making patients with an enlarged LVEDd more susceptible to SCD. This observation is consistent with other studies [18,19].

Other factors associated with a higher risk of arrhythmias leading to ATP or defibrillation, as indicated in the literature, include impaired kidney function [20], elevated NT-proBNP concentration [21], and a higher degree of HF symptoms assessed according to the NYHA scale. However, these findings were not confirmed in the present study. It is important to emphasize that the primary focus of this study was the analysis of parameters from cardiac adrenergic system scintigraphy and their impact on adequate ICD intervention.

The study also analyzed the impact of baseline parameters on mortality or heart transplantation.

As expected, older age was identified as a risk factor for this endpoint (*p* < 0.03). Additionally, a trend-level association was observed between the presence of diabetes at the time of ICD qualification and mortality or heart transplantation. This finding is supported by the literature, which explains the influence of diabetes on heart function and structure (microangiopathy of coronary vessels, metabolic alterations in cardiomyocytes, hypertension) [22].

Other variables that increased the risk of mortality or heart transplantation in the present study included higher NT-proBNP levels and lower resting heart rate. NT-proBNP is a parameter whose value logarithmically increases with the progression of heart failure (HF). Elevated NT-proBNP values reflect more advanced HF, which is undeniably associated with poorer prognosis. The resting heart rate, on the other hand, is influenced by the dose of beta-blockers, which were administered to all patients as part of optimal pharmacotherapy for HF. Thus, the observation of increased mortality risk in patients with a lower resting heart rate seems inconsequential.

Parameters indicated in the literature as associated with higher mortality risk but not confirmed in the present study include multiple episodes of myocardial infarction and a higher NYHA class [23,24].

Scintigraphic parameters did not effectively predict ICD interventions or death/heart transplantation, though the summed difference score demonstrated potential as a prognostic factor. In the univariate analysis, a high SDS value obtained in the early phase of scintigraphic examination was a predictor of death or heart transplantation (HR 1.079; 95% confidence interval 1.007–1.157; *p* = 0.0314). However, this was not confirmed in the multivariate analysis.

It is important to note the high percentage of low-quality SPECT images, resulting in a relatively small group included in the analysis (41 in the early phase, 47 in the late phase) [25]. Despite this, the SDS obtained in the early SPECT phase could potentially be a predictor of death or heart transplantation. Importantly, non-diagnostic SPECT images were more common in severe heart failure patients, where ICD implantation indications are less disputed. In this study, effective ICD therapy was observed in 74% of patients with EF 30–35% (*p* < 0.004), but only 11% with EF < 30% (*p* = 0.999), possibly due to higher mortality in the latter group (30% among patients with EF < 30%; *p* = 0.114). The less frequent occurrence of appropriate ICD intervention in patients with more advanced heart failure may be due to a higher incidence of the competing endpoint, namely death. Nevertheless, these observations emphasize that the group benefiting most from ICD implantation consists of patients with moderately impaired left ventricular systolic function.

This highlights the benefit of ICD implantation for patients with moderately impaired EF, aligning with multicenter trials like SCD-HeFT and MADIT II, which support ICD implantation for primary prevention in post-infarction heart failure patients with EF ≤ 35%, particularly for symptomatic individuals (NYHA II, III). Although the subgroup with EF 30–35% was small in these studies (20% in the SCD-HeFT study; not included in MADIT II), the overall evidence supports the benefit of ICD implantation in this group.

The 2022 ESC guidelines now also recommend electrophysiological studies (EPS) for patients with EF 35–40% and asymptomatic non-sustained ventricular tachycardia (NSVT) to assess potential ICD benefit. If complex arrhythmias are induced, ICD implantation should be considered (Class IIA Level B), reflecting a broader recognition of SCD risk and the need for ongoing non-invasive assessment in post-infarction heart failure patients.

Moreover, despite strong recommendations, the decision to qualify symptomatic patients with moderately impaired left ventricular systolic function for ICD implantation can be complex and debatable. This is often due to doubts about the precise assessment of EF, which remains a major challenge for echocardiographers. Calculating EF requires a precise outline of the endocardial line, which can be difficult and sometimes impossible (especially in obese patients or those with chronic obstructive pulmonary disease, emphysema, or post-thoracotomy). Given the consequences of incorrect EF assessment and the associated potential risk of misclassifying patients for ICD implantation, having an additional method to evaluate SCD risk would be highly valuable in clinical practice.

Despite its limitations, SPECT imaging using [^123^I]MIBG could potentially serve this purpose. However, this requires analysis of a larger patient cohort. Interestingly, evidence in the literature suggests that in a more narrowly defined group of patients, specifically those qualified for CRT-D, 123-I-metaiodobenzylguanidine (^123^I-mIBG) imaging has shown potential for distinguishing resynchronization therapy responders from non-responders [26]. Available data also suggest that the H/M ratio is inversely proportional to LVEF, a recognized predictor of cardiac events [27]. In patients with extensive myocardial damage, a lower H/M ratio and worse prognosis are expected. Independent researchers are testing his hypothesis and the use of the H/M ratio to predict cardiac events. Preliminary results have not consistently shown that the H/M ratio is a strong independent predictor of arrhythmias, recurrent myocardial infarction, heart failure hospitalizations, or death. However, a meta-analysis of 18 studies involving 1755 patients demonstrated that lower early or late H/M values were associated with higher rates of death, recurrent MI, or heart failure hospitalizations [28]. Another scintigraphic parameter with potential predictive value for events such as ICD interventions and death is the summed defect score (SDS). A 2010 study by Boogers et al. analyzed 116 heart failure patients and found that those with high SDS values experienced significantly more ICD interventions and higher mortality [29].

This highlights the need to explore this area further and conduct additional studies to deepen understanding and improve risk stratification. Although the SPECT imaging using [^123^I]MIBG did not prove helpful in the cohort analyzed in this study, the observations made after additional analysis of all the data suggest how a well-designed study could provide valuable information regarding the prediction of life-threatening arrhythmias and mortality.

Additional verification would be most valuable for patients with borderline indications for ICD implantation. Therefore, it is worth considering narrowing the analyzed group to patients with EF 30–35% and possibly including those with EF 35–40%.

## 5. Study Limitation

A key limitation of this study is its relatively small cohort size, which is partly due to it being a single-center study. This smaller sample is also a factor compared to studies that use data from medical interviews, basic laboratory tests, or echocardiographic examinations.

Another limitation is that the study was designed in 2013, with patient enrollment occurring from 2014 to 2017, based on the previous ESC guidelines for the treatment of heart failure. As a result, treatment protocols were aligned with the standards applicable during that period.

Additionally, the study was conducted at the National Institute of Cardiology, a tertiary care center. Such centers often treat patients with more advanced heart disease and complex conditions, which may lead to worse prognoses compared to the general population. Clinical trials typically involve patients with fewer comorbidities and are conducted under controlled conditions, which may not fully reflect real-world clinical practice. These differences might not affect the overall prognosis but could influence how various factors impact endpoints, such as ICD intervention or death.

Furthermore, it is essential to emphasize that the primary focus of the study was to evaluate the impact of cardiac adrenergic system scintigraphy. Consequently, the lack of a demonstrated correlation between scintigraphic parameters and mortality might be a Type I error.

## 6. Conclusions

The analyzed scintigraphic parameters did not prove useful in predicting ICD interventions or mortality. The low-quality SPECT images in patients with low LVEF resulted in only 62% of patients being included in the analysis. Despite this limitation, the univariate analysis highlighted the significance of the SDS parameter as a predictor of poor prognosis. The substantial rate of appropriate ICD interventions in patients with EF 30–35% and those with moderately impaired LV function underscores the need for further in-depth analysis in this patient subgroup.

## Figures and Tables

**Table 1:** Summary of Inclusion and Exclusion Criteria.

Inclusion Criteria	Exclusion Criteria
Ejection fraction (EF) ≤ 35% despite at least 3 months of optimal medical therapy	Age ≤ 50 years
NYHA functional class II or III	≤3 months post-revascularization (PCI, CABG)
Reasonable expectation of survival with good functional status for >1 year	Post-ICD/CRT-D implantation
At least 40 days post myocardial infarction	Allergy to iodine
	Planned coronary revascularization
	Lack of conscious, written consent to participate in the study.

**Table 2:** Clinical characteristics of patients—analyzed variable ATP/defibrillation.

Parameter		All Patients (n = 85)	No Event (n = 66)	ATP or Defibrillation (n = 19)	*p*-Value
Age, mean ± SD		66.4 ± 8.4	67.8 ± 7.8	61.3 ± 8.5	0.0022
Men, n (%)		74 (87.06)	56 (84.85)	18 (94.74)	0.4425
NYHA					
	II, n (%)	58 (74.36)	46 (76.67)	12 (66.67)	0.5387
	III/IV, n (%)	20 (25.64)	14 (23.33)	6 (33.33)	
Primary prevention of SCD, n (%).		76 (89.41)	57 (86.36)	19 (100.00)	0.1977
Nicotine, n (%)		28 (34.15)	22 (33.85)	6 (35.29)	0.9108
Hypertension, n (%)		51 (60.71)	41 (63.08)	10 (52.63)	0.4122
Diabetes, n (%)		22 (25.88)	16 (24.24)	6 (31.58)	0.5586
Number of myocardial infarctions					
	1	56 (70.00)	44 (68.75)	12 (75.00)	0.7652
	≥2	24 (30.00)	20 (31.25)	4 (25.00)	
Number of PCI procedures					
	0	21 (26.25)	17 (26.56)	4 (25.00)	0.0880
	1	36 (45.00)	32 (50.00)	4 (25.00)	
	≥2	23 (28.75)	15 (23.44)	8 (50.00)	
CABG, n (%)		17 (21.25)	12 (18.75)	5 (31.25)	0.3117
EF, Me (IQR)		28 (25–30)	25.5 (25–30)	30 (28–34)	0.0537
EF ≥ 30 (%), n (%)		38 (44.71)	24 (36.36)	14 (73.68)	0.0039
EF < 25 (%), n (%)		17 (20.00)	13 (19.70)	4 (21.05)	0.9999
LVEDd (mm) mean ± SD		64.3 ± 7.1	63.7 ± 6.8	66.5 ± 7.6	0.1373
HR (bpm), mean ± SD		69.0 ± 10.8	68.7 ± 10.7	70.0 ± 11.4	0.6252
lnNT-proBNP, Me (IQR)		7.0 (6.5–7.8)	7.1 (6.6–7.9)	6.8 (6.3–7.3)	0.1747
creatinine (mmol/L), Me (IQR)		102 (81–118)	100 (79–118)	103 (82–126)	0.4849
H/M-15′, Me (IQR)		1.7 (1.5–1.9)	1.7 (1.5–1.9)	1.8 (1.5–1.8)	0.9011
H/M-4h, Me (IQR)		1.5 (1.3–1.7)	1.5 (1.3–1.7)	1.5 (1.4–1.7)	0.9419
WO (%), mean ± SD		38.8 ± 16.8	39.1 ± 17.6	37.7 ± 14.0	0.7449
SDS-15′, mean ± SD		29.2 ±10.7	28.2 ± 11.4	32.4 ± 7.4	0.2737
SDS-4h, mean ± SD		33.4 ± 10.2	32.4 ± 10.4	36.3 ± 9.3	0.2189
Observation period (years), Me (IQR)		4.0 (2.0–5.7)	4.4 (2.1–6.1)	2.3 (1.2–5.2)	0.0194

SD—Standard Deviation; Me—Median; IQR—Interquartile Range; n—Number of patients; ATP—Antitachyarrhythmic Pacing; NYHA—New York Heart Association Heart Failure Classification; SCD—Sudden Cardiac Death; PCI—Percutaneous Coronary Intervention; CABG—Coronary Artery Bypass Grafting; EF—Ejection Fraction; LVEDd—Left Ventricular End-Diastolic Dimension; mm—millimeter; HR—Heart Rate; lnNT-proBNP—Natural Logarithm of N-Terminal pro-B-Type Natriuretic Peptide concentration; H/M-15′—Heart-to-Mediastinum Ratio in early planar imaging; H/M-4h—Heart-to-Mediastinum Ratio in late planar imaging; WO—Washout Rate; SDS-15′—Summed Defect Score in early SPECT imaging; SDS-4h—Summed Defect Score in late SPECT imaging.

**Table 3:** Clinical characteristics of patients—analyzed variable: death.

Parameter		All Patients (n = 85)	No Event (n = 64)	Death or Heart Transplant (n = 21)	*p*-Value
Age (lat), mean ± SD		66.4 ± 8.4	65.4 ± 7.8	69.3 ± 9.5	0.0840
Men, n (%)		74 (87.06)	55 (85.94)	19 (90.48)	0.7241
NYHA					
	II, n (%)	58 (74.36)	46 (75.41)	12 (70.59)	0.7563
	III/IV, n (%)	20 (25.64)	15 (24.59)	5 (29.41)	
Primary prevention of SCD, n (%)		76 (89.41)	58 (90.63)	18 (85.71)	0.6832
Nicotine, n (%)		28 (34.15)	24 (38.71)	4 (20.00)	0.1250
Hypertension, n (%)		51 (60.71)	38 (59.38)	13 (65.00)	0.6530
Diabetes, n (%)		22 (25.88)	14 (21.88)	8 (38.10)	0.1409
Number of myocardial infarctions					
	1	56 (70.00)	42 (68.85)	14 (73.68)	0.6882
	≥2	24 (30.00)	19 (31.15)	5 (26.32)	
Number of PCI procedures					
	0	21 (26.25)	15 (24.59)	6 (31.58)	0.7021
	1	36 (45.00)	29 (47.54)	7 (36.84)	
	≥2	23 (28.75)	17 (27.87)	6 (31.58)	
CABG, n (%)		17 (21.25)	11 (18.03)	6 (31.58)	0.2158
EF, Me (IQR)		27.2 ± 5.3 28 (25–30)	27.7 ± 5.4 28 (25–30)	25.6 ± 4.5 25 (20–30)	0.1045
EF ≥ 30 (%), n (%)		38 (44.71)	31 (48.44)	7 (33.33)	0.2270
EF < 25 (%), n (%)		17 (20.00)	10 (15.62)	7 (33.33)	0.1140
LVEDd (mm) mean ± SD		64.3 ± 7.1	64.2 ± 7.0	64.5 ± 7.3	0.7758
HR (bpm), mean ± SD		69.0 ± 10.8	70.4 ± 11.1	63.6 ± 8.2	0.0094
lnNT-proBNP, Me (IQR)		7.0 (6.5–7.8)	6.9 (6.4–7.5)	7.4 (6.9–8.2)	0.0122
creatinine (mmol/L), Me (IQR)		102 (81–118)	95 (80–118)	106 (82–120)	0.4086
H/M-15′, Me (IQR)		1.7 (1.5–1.9)	1.7 (1.5–1.9)	1.8 (1.5–1.9)	0.1621
H/M-4h, Me (IQR)		1.5 (1.3–1.7)	1.5 (1.3–1.7)	1.5 (1.3–1.7)	0.8301
WO (%), mean ± SD		38.8 ± 16.8	37.9 ± 18.0	41.7 ± 12.4	0.2851
SDS-15′, mean ± SD		29.2 ± 10.7	27.0 ± 11.0	34.8 ± 7.8	0.0580
SDS-4h, mean ± SD		33.4 ± 10.2	32.6 ± 11.0	35.7 ± 7.0	0.4821
Observation period (years), Me (IQR)		4.8 (2.6–6.1)	5.2 (3.8–6.3)	2.1 (1.3–2.8)	<0.0001

SD—Standard Deviation; Me—Median; IQR—Interquartile Range; n—Number of patients; NYHA—New York Heart Association Heart Failure Classification; SCD—Sudden Cardiac Death; PCI—Percutaneous Coronary Intervention; CABG—Coronary Artery Bypass Grafting; EF—Ejection Fraction; LVEDd—Left Ventricular End-Diastolic Dimension; mm—millimeter; HR—Heart Rate; lnNT-proBNP—Natural Logarithm of N-Terminal pro-B-Type Natriuretic Peptide concentration; H/M-15′—Heart-to-Mediastinum Ratio in early planar imaging; H/M-4h—Heart-to-Mediastinum Ratio in late planar imaging; WO—Washout Rate; SDS-15′—Summed Defect Score in early SPECT imaging; SDS-4h—Summed Defect Score in late SPECT imaging.

**Table 4:** Clinical events in patients.

Event	Number of Patients (%)
Appropriate defibrillation	11 (13)
Appropriate ATP therapy	8 (9)
Death	20 (24)
Heart transplant	1 (1)

ATP—Antitachyarrhythmic Pacing.

**Table 5:** Analysis of factors influencing the occurrence of ICD intervention or death/heart transplant. Univariate Cox analysis.

	Death or Heart Transplant		ATP or Defibrillation	
Parameter	HR (95% CI)	*p*-Value	HR (95% CI)	*p*-Value
Age (years)	1.065 (1.007–1.126)	0.0263	0.894 (0.829–0.964)	0.0036
Men	1.423 (0.331–6.118)	0.6359	3.717 (0.494–27.960)	0.2022
NYHA	0.818 (0.287–2.328)	0.7066	0.639 (0.239–1.708)	0.3721
Primary prevention of SCD	1.853 (0.543–6.321)	0.3242	-	-
EF	0.933 (0.864–1.007)	0.0742	1.042 (0.954–1.139)	0.3586
EF ≥ 30%	0.502 (0.202–1.249)	0.1387	3.125 (1.122–8.701)	0.0292
EF ≥ 25%	0.387 (0.155–0.964)	0.0415	0.810 (0.267–2.453)	0.7092
EF < 25%	2.587 (1.038–6.452)	0.0415	1.235 (0.408–3.740)	0.7092
LVEDd (mm)	1.010 (0.948–1.076)	0.7657	1.074 (1.004–1.150)	0.0384
Nicotine	0.493 (0.165–1.476)	0.2063	1.136 (0.420–3.076)	0.8011
Hypertension	1.149 (0.457–2.886)	0.7682	0.629 (0.254–1.554)	0.3148
Diabetes	2.224 (0.914–5.414)	0.0782	1.538 (0584–4.054)	0.3836
Number of myocardial infarctions				
1	1	-	-	-
2	0.492 (0.111–2.168)	0.3482	-	-
3	2.212 (0.632–7.737)	0.2139	-	-
Number of myocardial infarctions				
≥2	0.922 (0.331–2.566)	0.8760	0.917 (0.295–2.848)	0.8813
Number of PCI procedures				
0	1	-	1	-
1	0.715 (0.240–2.131)	0.5470	0.591 (0.147–2.367)	0.4572
≥2	1.017 (0.327–3.161)	0.9765	2.186 (0.657–7.273)	0.2024
PCI	0.828 (0.314–2.183)	0.7034	1.153 (0.372–3.578)	0.8054
CABG	1.847 (0.701–4.870)	0.2146	2.278 (0.789–6.575)	0.1281
lnNT-proBNP	1.795 (1.167–2.763)	0.0078	0.830 (0.531–1.297)	0.4127
Creatinine mmol/L	1.009 (0.998–1.020)	0.1095	1.005 (0.992–1.018)	0.4440
HR (bpm)	0.922 (0.861–0.987)	0.0193	1.001 (0.955–1.049)	0.9775
H/M-15′	2.599 (0.455–14.857)	0.2829	0.784 (0.135–4.557)	0.7865
H/M-4h	1.034 (0.152–7.043)	0.9731	0.639 (0.087–4.684)	0.6591
SDS-15′	1.079 (1.007–1.157)	0.0314	1.055 (0.990–1.124)	0.1018
SDS-4h	1.038 (0.976–1.104)	0.2344	1.048 (0.986–1.115)	0.1319
H/M-15′ > 1.6	1.527 (0.586–3.982)	0.3866	1.368 (0.519–3.607)	0.5259
H/M-4h > 1.6	0.726 (0.287–1.837)	0.4993	0.464 (0.166–1.297)	0.1434
H/M-15′ > 1.73	2.066 (0.823–5.186)	0.1221	1.465 (0.587–3.651)	0.4130
H/M-4h > 1.73	1.030 (0.373–2.846)	0.9545	0.564 (0.164–1.940)	0.3634
WO (%)	1.015 (0.991–1.040)	0.2208	1.002 (0.975–1.030)	0.8803

HR—Hazard Ratio; CI—Confidence Interval for HR; ATP—Antitachyarrhythmic Pacing; NYHA—New York Heart Association Heart Failure Classification; PCI—Percutaneous Coronary Intervention; CABG—Coronary Artery Bypass Grafting; EF—Ejection Fraction; LVEDd—Left Ventricular End-Diastolic Dimension; mm—millimeter; HR—Heart Rate; lnNT-proBNP—Natural Logarithm of N-Terminal pro-B-Type Natriuretic Peptide concentration; H/M-15′—Heart-to-Mediastinum Ratio in early planar imaging; H/M-4h—Heart-to-Mediastinum Ratio in late planar imaging; WO—Washout Rate; SDS-15′—Summed Defect Score in early SPECT imaging; SDS-4h—Summed Defect Score in late SPECT imaging.

**Table 6:** Analysis of factors influencing the occurrence of death or heart transplant. Multivariate Cox analysis.

Parameter	HR (95% CI)	*p*-Value
Age	1.062 (1.006–1.122)	0.0309
EF < 25%	2.512 (1.004–6.286)	0.0490

HR—Hazard Ratio; CI—Confidence Interval for HR; EF—Ejection Fraction.

**Table 7:** Analysis of factors influencing the occurrence of ICD intervention. Multivariate Cox analysis.

Parameter	HR (95% CI)	*p*-Value
Age	0.912 (0.846–0.983)	0.0162
EF ≥ 30%	4.632 (1.538–13.954)	0.0064
LVEDd (mm)	1.096 (1.017–1.181)	0.0164

HR—Hazard Ratio; CI—Confidence Interval for HR; EF—Ejection Fraction; LVEDd—Left Ventricular End-Diastolic Dimension; mm—millimeter.

**Table 8:** Analysis of H/M ratio values as predictors for the occurrence of death/ATP or defibrillation in the literature.

Publication	Parameter	All Patients	No Event	Death or Heart Transplant	*p*-Value	No Event	ATP or Defibrillation	*p*-Value
A. F. Jacobson et al., *J. Am. Coll. Cardiol.* **55**, 2212–2221 (2010) [11]	H/M-4h > 1.6, n (%)	31 (36.90)	24 (37.50)	7 (35.00)	0.8397	26 (40.00)	5 (26.32)	0.2769
T. Arimoto et al., *J. Card. Fail.* **13**, 34–41 (2007) [12]	H/M-4h > 1.73, n (%)	19 (22.62)	14 (21.88)	5 (25.00)	0.7660	16 (24.62)	3 (15.79)	0.5420
D. Agostini et al., *Eur. J. Nucl. Med. Mol. Imaging* **35**, 535–546 (2008) [13]	H/M-4h < 1.45	32 (38.10)	24 (37.50)	8 (40.00)	0.8407	25 (38.46)	7 (36.84)	0.8983
D. O. Verschure et al., *Int. J. Cardiol.* **248**, 403–408 (2017) [14]	H/M-4h 1.4–2.1	The sample size is too small to conduct a reliable analysis.						
M. I. Travin et al., *J. Nucl. Cardiol.* **24**, 377–391 (2017) [15]	H/M 1.3–1.59							
D. Agostini et al., *Eur. J. Nucl. Med. Mol. Imaging* **35**, 535–546 (2008) [13]	H/M-4h > 1.75, n (%)	18 (21.43)	13 (20.31)	5 (25.00)	0.7562	15 (23.08)	3 (15.79)	0.7514

H/M-4h—Heart-to-Mediastinum Ratio in late planar imaging; ATP—Antitachyarrhythmic Pacing.

## Data Availability

The data presented in this study are available on request from the corresponding author. The data are not publicly available due to privacy.
